# Promoting an obesity education program among minority patients in a single urban pediatric Emergency Department (ED)

**DOI:** 10.1186/s12245-015-0086-z

**Published:** 2015-10-28

**Authors:** Jordana J. Haber, Sukshant Atti, Linda M. Gerber, Muhammad Waseem

**Affiliations:** Department of Emergency Medicine, University Medical Center, 1800 W Charleston Blvd, Las Vegas, NV 89102 USA; Department of Emergency Medicine, Lincoln Medical & Mental Health Center, 234 E 149th St, Bronx, NY 10451 USA; Department of Healthcare Policy, Research Weill Cornell Medical College, 402 East 67th Street, New York, NY 10065 USA

## Abstract

**Background:**

The purpose of this study was to assess the feasibility of the Emergency Department (ED) as a place for obesity education and to evaluate its impact on patient’s lifestyle modification.

**Methods:**

In this study, children between 8 and 18 years of age, who presented to the ED for non-urgent reasons in a single urban hospital, were enrolled. Parents’ perception of their child’s diet and exercise were assessed prior to the intervention. Both parents and children attended a brief audio-visual presentation that provided educational information on age-appropriate diet and exercise. Following the intervention, the participants were asked about their impressions regarding the ED as a place to receive obesity education and whether they plan to make any changes in diet and exercise.

**Results:**

One hundred children and their parents participated in this study. Of these, 76 were Latino and 21 were African-Americans. The mean age was 14 years, and the mean body mass index (BMI) was 25.6. Following the intervention, 21 (100 %) of the African-American parents and 73 (98.6 %) of the Latino parents felt that the ED should provide obesity education. Eighteen (85.7 %) of the African-American parents and 72 (97.3 %) of the Latino parents planned to make changes in their child’s diet and exercise. Among the children, 21 (100 %) of African-American participants and 76 (100 %) of Latino participants reported that they found the audio-visual useful. Seventeen (81.0 %) of the African-American children and 73 (96.1 %) of Latino children stated learning new information from the intervention program.

**Conclusions:**

This study suggests the ED may have a role in primary health promotion and obesity prevention. An ED-based intervention may be used to provide education about obesity prevention and has the potential to impact life style modifications, including diet and exercise.

## Background

The prevalence of obesity has risen dramatically in the USA, with the rate increasing more than threefold during the past three decades [1–3]. The prevalence of overweight individuals has increased from 6 % (in 1980) to 19 % (2004) [4, 5]. The most alarming is that childhood obesity most often will continue into adulthood [6]. The consequences of obesity include coronary artery disease, diabetes, adverse psychological problems, and premature morbidly and mortality [7–9]. In addition, if not addressed, studies predict large societal costs associated with the obesity epidemic [10, 11].

Interventions to date have targeted childhood obesity-related habits, such as sedentary lifestyle and over-consumption of high-energy food containing empty calories. Follow-up studies of prior intervention programs show that interventions focused on education, lifestyle modification, and exercise can positively influence both food choices and exercise activity for up to 1 year [12]. When successful in reducing obesity prevalence, these simple interventions are well worth the investment in return for an improved quality of life and a decrease in the health and societal costs directly associated with obesity [13].

In many communities, the hospital Emergency Department (ED) is a major source of primary care [14]. We hypothesize that the ED can serve as a place for effective intervention and education in the obesity epidemic. Although many intervention programs are found in the literature, the authors were unable to find a report of ED-based intervention. The implication, if this hypothesis is true, would be to encourage ED physicians to address patient obesity and to initiate intervention planning.

Evaluating the ED as an effective place for intervention in childhood and adolescent obesity is important for at least two reasons: First, such a study is consistent with the national health initiative, Healthy People 2020. Listed among their goals are reducing health inequalities, increasing the proportion of physician visits which provide counseling and education related to nutrition and weight. Obesity has been identified as one of the 10 leading health indicators for the nation, and curbing the growing epidemic of childhood and adolescent obesity has become an important national priority [1]. As part of a multidisciplinary approach to combat the obesity epidemic, the ED serves as an ideal place to provide intervention.

Second, the prevalence of overweight individuals and obesity is disproportionately higher among urban youth, with the highest rate affecting minority groups [1]. There is a substantially higher prevalence of obesity found among Latino boys and non-Latino black girls [15]. In addition, the increase in obesity (children with a body mass index (BMI) >99th percentile for the age) is common among Latino, black, and disadvantaged children [16]. Thus, targeting intervention efforts toward minority youth has the potential to reduce health disparities across multiple disease conditions. This study was conducted at a high-volume inner-city hospital that serves a high percentage of minority youth.

We designed a prospective study at our pediatric urban community ED to test whether it was feasible and of value to enroll patients and their parents in an educational Obesity Intervention Program (OIP), as a means of prevention and health education. The aim of this study was to evaluate participants’ perceptions of receiving obesity related education and assess the effectiveness of an obesity educational sessions in an urban pediatric ED.

## Methods

This was a prospective study conducted at a single pediatric ED of an inner-city teaching hospital. This study was approved by the institutional review board of Lincoln Hospital. We enrolled 100 children from 8 to 18 years of age, and their parents, who presented to the ED for non-urgent reasons between August 2011 and June 2012. Enrollment periods and times were at the convenience of the research team and were always conducted during weekday working hours. Written informed consent was obtained from the patient for the publication of this report and any accompanying images

All participants and their parents were given a self-administered perception questionnaire prior to the intervention. The parents’ perception of their child’s health was assessed by asking if they believed their child was at an appropriate weight, if their child exercised enough, as well as their perception of the child’s overall eating habits.

Once the questionnaire was completed, the participants were escorted to a room in the ED and were shown a 5-min audio-visual educational intervention presentation, adopted from the National Institute of Health (NIH) website (http://kidshealth.org/kid/stay_healthy/fit/fit_kid.html#) to provide educational information on age-appropriate diet and exercise regimens. The video, available in both Spanish and English, was intended to educate the general population.

We recorded demographic characteristics, and BMIs were calculated based on the height and weight measurements obtained in the ED for each child. The BMIs of the enrolled children were organized into underweight, normal weight, overweight, and obese groups. These groups were based on the following NIH BMI categories: (a) less than 18.5, underweight; (b) between 18.5 and 24.9, normal weight; (c) between 25 and 29.9, overweight; and (d) obese, 30 or greater

Following the video viewing, the participants were asked to complete a self-administered questionnaire to assess their opinions regarding the ED as a place to receive obesity education and whether the parents planned to make any changes in their child’s diet and exercise regimens.

## Results

One hundred parents and their children were surveyed. The mean age of the children was 14 years (8–18 years old), and the mean BMI for the children was 25.6.

**Fig. 1:**
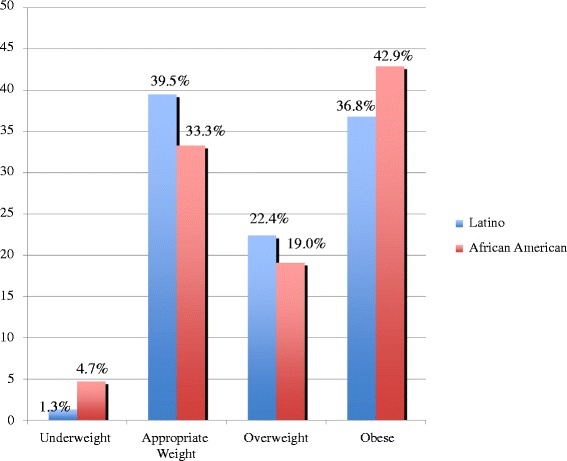
Distribution of weight categories by ethnicity according to BMI categories

**Fig. 2:**
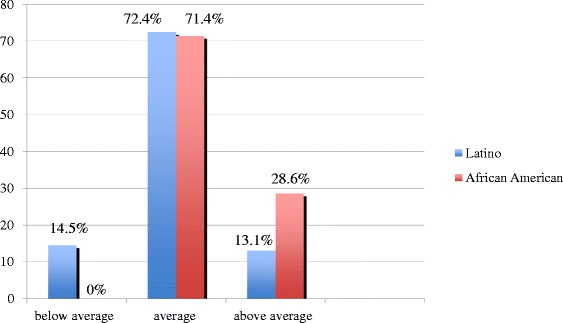
Parent’s perception of child’s fitness

Of these, 76 were Latino, 21 were African-American, and 3 were listed as “other”. Due to the small sample size of children in the “other” category, the analyses were limited to the 97 Latino and African-American participants. This sample size is representative of the patient population at this hospital. Among the Latino children, 1 (1.3 %) child was underweight, 30 (39.5 %) had age-appropriate weight, 17 (22.4 %) were overweight, and 28 (36.8 %) were obese. Among the 21 African-American children, 1 (4.7 %) child was underweight, 7 (33.3 %) had age-appropriate weight, 4 (19.0 %) were overweight, and 9 (42.9 %) were obese (Fig. 1). When asked about their children’s fitness, among the Latino parents, 55 (72.4 %) felt that their children were of average fitness, 10 (13.1 %) felt they were above average, and 11 (14.5 %) felt that their children were below average in fitness. Fifteen (71.4 %) of the African-American parents felt their children were of average fitness, 6 (28.6 %) felt their children had above average fitness, and none felt they were below average fitness level (Fig. 2).

**Fig. 3:**
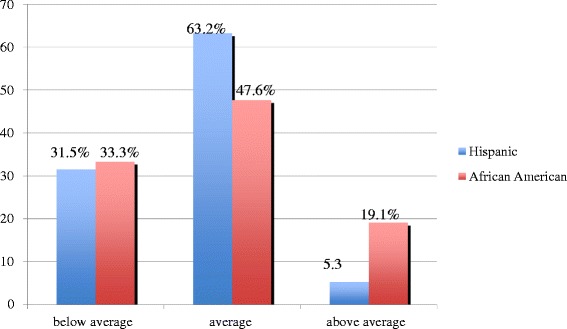
Parent’s perception of child’s diet

Among the Latino parents, 24 (31.5 %) felt that their child had a below average healthy diet, 48 (63.2 %) reported their child had an average healthy diet, and 4 (5.3 %) had an above average healthy diet. Among the African-American parents, 7 (33.3 %) felt that their child had a below average healthy diet, 10 (47.6 %) had an average healthy diet, and 4 (19.1 %) had an above average healthy diet (Fig. 3).

**Fig. 4:**
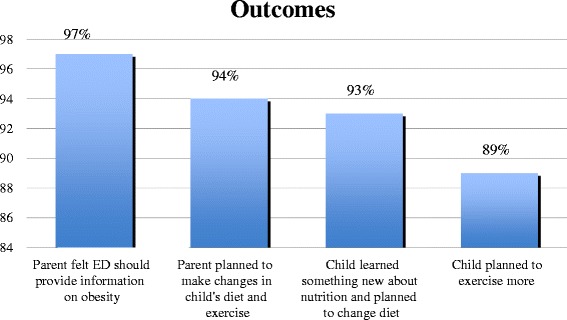
Parent and child perceptions of an ED obesity education program

In the parent populations, 21 (100 %) of the African-American participants and 73 (96.1 %) of the Latino participants found the information in the audio-video presentation to be useful. Twenty-one (100 %) of the African-American parents and 73 (98.6 %) of the Latino parents felt the ED should offer information about obesity and healthy lifestyle. Eighteen (85.7 %) of the African-American parents and 72 (97.3 %) of the Latino parents reported that they planned to make changes in their child’s exercise and diet regimens after the ED intervention. Among the children, 21 (100 %) of African-American participants and 76 (100 %) of Latino participants reported that they found the video useful. Seventeen (81.0 %) of the African-American children and 73 (96.1 %) of the Latino children reported learning something new about nutrition and exercise from the intervention program. Seventeen (81 %) of the African-American children and 73 (96.1 %) of the Latino children reported they planned to change what they eat 17 (81.0 %), and 69 (90.8 %) respectively stated that they planned to exercise more following what they had learned during the intervention program (Fig. 4).

## Discussion

Childhood obesity is an increasing concern and is most pronounced among urban and lower socio-economic groups. The consequence, if not addressed, is a higher prevalence of chronic disease, associated medical emergencies, as well as social, psychological, and societal costs relating to the consequences of obesity. Medical complications of obesity during childhood include (a) a higher incidence of asthma [17] and other respiratory issues, such as sleep apnea [18, 19]; (b) abdominal emergencies [20, 21]; (c) anesthesia complications during surgeries [22, 23]; (d) orthopedic emergencies [24, 25]; and (e) childhood hypertension [26].

Current interventions should aim to decrease obesity, specifically in communities with the greatest health disparity. Data from the National Center for Health Statistics estimates that 17 % of children and adolescents between 2 and 19 years of age can be classified as obese according to BMI calculations [2, 3]. Our sample demonstrated a much higher proportion of obesity than the national average. Medical systems have responded to the obesity epidemic with increased weight and health evaluations, for earlier identification and counseling focusing on healthy lifestyle.

This study was developed to investigate feasibility and perception of a health and obesity education intervention program in a single high-volume urban pediatric ED. Our sample size included 100 children and their parents. Because the majority of the patients seen in our hospital were of minority background, we focused on this population in our study. While it is clear that obesity is a public health concern, less is known about the best way to treat the obesity epidemic. Research suggests that interventions are most effective when parents are involved [27]. In our intervention program, both parent and children attended an interactive session together. We accounted for both children and parent perceptions in our study. Specifically, we were interested to determine if participants perceived the ED as an appropriate and effective place for receiving information on obesity and healthy lifestyle habits. In our study, a majority of participants reported that the ED was an appropriate place to receive this information and planned to make changes for a healthier life style.

Limitations of our study included a single hospital, small population size, and a self-selection bias among the participants who enrolled in the study. This study included 100 participants. So far, the results were significant at this level, and a larger sample size was unlikely to yield different results. In addition, this study focused on a minority population. It is possible that a different population would have yielded different results. Further follow-up studies should include a larger sample size and multiple centers. This study was envisioned as a pilot study to see if a health intervention program in our pediatric ED was feasible and might be well-received by participants.

## Conclusions

This study suggests that parents and their children are interested in receiving education addressing healthier lifestyle choices and they also feel that the ED is an appropriate setting for such an intervention program. While a comprehensive approach with community, schools, and policies might be more effective, this study indicates that the ED may have a role in introducing and instructing patients about these lifestyle changes. Further studies should be employed to address long-term outcomes and should include objective endpoints.
